# Mapping tipping risks from Antarctic ice basins under global warming

**DOI:** 10.1038/s41558-025-02554-0

**Published:** 2026-02-16

**Authors:** Ricarda Winkelmann, Julius Garbe, Jonathan F. Donges, Torsten Albrecht

**Affiliations:** 1 Earth Resilience Science Unit, Potsdam Institute for Climate Impact Research (PIK), Member of the Leibniz Association, Potsdam, Germany; 2 Integrative Earth System Science, Max Planck Institute of Geoanthropology, Jena, Germany; 3 Institute of Physics and Astronomy, University of Potsdam, Potsdam, Germany; 4 Stockholm Resilience Centre, Stockholm University, Stockholm, Sweden

**Keywords:** Cryospheric science, Projection and prediction, Climate-change impacts

## Abstract

The Antarctic Ice Sheet is subject to amplifying feedbacks which can accelerate ice loss and lead to effectively irreversible retreat. We here analyse the distinct nature and risk of long-term ice loss for each individual drainage basin under different levels of warming. Depending on topographic and climatic conditions, we find that ice loss in some basins unfolds gradually with warming, whereas other basins are characterized by a critical threshold or tipping point beyond which large parts eventually disintegrate. A first threshold, potentially as low as 1–2 °C above pre-industrial levels, triggers the long-term collapse of ~40% of marine ice volume in West Antarctica. Marine-based sectors in East Antarctica, representing ~5 m of potential sea-level rise, are at risk of losing stability at 2–5 °C. Our results imply that the Antarctic Ice Sheet does not act as one single tipping element, but rather as several tipping systems interacting across drainage basins.

## Abstract (web summary)

Climate change threatens the future of the Antarctic Ice Sheet. Here the authors show that individual drainage basins have different thresholds and loss patterns, suggesting the need to consider the dynamical interactive nature of the basins and their individual tipping points.

## Main

The Antarctic Ice Sheet is the largest ice sheet on Earth with a mass equivalent to nearly 60 m of global sea-level rise potential^1^. Its stability and future response to a warming climate is therefore highly relevant for coastal communities, infrastructure and ecosystems^2^. Under future anthropogenic climate change, the ice sheet is ‘projected to lose mass at an increasing rate throughout the twenty-first century and beyond (high confidence)’^2^, which could commit future generations to long-term sea-level rise^3,4^, with subsequent impacts including coastal erosion, ecosystem loss, human livelihood and infrastructure displacement, increased hazards from storm surges and potential groundwater salinification^5^. Ice loss from Antarctica would also affect the Southern Ocean and could lead to a weakening of Antarctic bottom water formation^6^, which would have cascading effects on the global ocean and climate^7–9^.

Palaeorecords and modelling suggest that Antarctica has undergone periods of large-scale and abrupt ice loss in the past^10–13^. During past interglacial warm periods that were only slightly (~1–3 °C) warmer than today despite broadly comparable (~300–400 ppm) atmospheric CO_2_ concentrations, the Antarctic Ice Sheet probably contributed several metres to global sea level^14,15^, implying substantial retreat of marine ice-sheet regions in both West^12,16,17^ and East Antarctica^18–20^. In particular, meltwater pulses due to accelerated ice-sheet retreat in Antarctica during the last glacial termination might have caused sea levels to rise at rates of up to ~0.7 m per century (or ~7 mm yr^−1^) (ref. ^21^).

On the basis of these palaeoreconstructions as well as modelling studies and process understanding, the Antarctic Ice Sheet is deemed a tipping element in the climate system^22–25^. This means that beyond a critical threshold (or several thresholds), self-sustaining feedbacks can lead to abrupt and often irreversible ice loss, with far-reaching impacts on the Earth system via global sea-level rise and changes in atmospheric and oceanic conditions and circulation patterns.

Observations indicate that in particular the West Antarctic Ice Sheet has been losing mass at an accelerating pace over the last decades, leading to increasing contributions to global mean sea-level rise^26,27^. The Amundsen Sea Embayment sector in West Antarctica shows first signs of destablization in response to ocean-induced thinning that reduces ice-shelf buttressing^28–30^. Also, in Wilkes Land in East Antarctica, increased ice discharge has been observed in response to recent warming^31^.

While palaeoreconstructions and climate modelling suggest that snowfall in Antarctica will probably increase with global warming^32–34^—which can mitigate some of the expected ice loss^35,36^—enhanced ablation, dynamical losses and amplifying feedbacks will probably dominate the overall mass balance in the future^37,38^.

Among the most prominent amplifying feedbacks are the surface melt–elevation feedback^39,40^, the melt–albedo feedback^41^, the marine ice-sheet instability^42,43^ (MISI) and the potential marine ice cliff instability^11,44,45^ (MICI); and further amplifying feedbacks have been suggested^46–48^. As surface melt is still very limited in Antarctica because of the cold surface temperatures, the melt–elevation and melt–albedo feedbacks are probably going to become more relevant under future, considerably warmer, conditions^49^. Current mass loss is dominated by ocean-driven subshelf melting^50–53^ and iceberg calving^51,54^. In marine ice-sheet regions, where the ice rests on bedrock below sea level, this can trigger MISI, an amplifying feedback between grounding-line retreat and the ice flux across the grounding line^42,43^. In fact, recent studies suggest that parts of the Amundsen Sea Embayment region in West Antarctica might already be undergoing unstable retreat^55–58^ or that large-scale irreversible ice loss might be imminent^59,60^. Other subglacial basins in East Antarctica are also at risk of undergoing rapid ice loss due to MISI^61,62^. If MICI, the mechanical failure and consequent self-perpetuating collapse of tall ice cliffs^44^, were to be triggered, this would further increase the potential of abrupt ice loss^11,63^. However, observational constraints on the related processes are limited and large uncertainties remain regarding the imposed risks^64,65^. Moreover, the conditions and potential stabilizing processes, such as mélange buttressing^66^ or glacial isostatic adjustment^67,68^, might counteract these instability mechanisms. Overall, the future long-term evolution of the Antarctic Ice Sheets depends on the complex interplay of these amplifying and dampening feedbacks, as well as its interactions with other parts of the global climate system^69–71^.

Future projections of contributions of the Antarctic Ice Sheet to sea-level rise hence involve large uncertainties^38,72^: While projections with multiple models range from 3 cm to 34 cm (likely range; 11–12 cm median, depending on shared socioeconomic pathway (SSP) scenario) global sea-level contribution from Antarctica by 2100 relative to 1995–2014^5^, uncertainties increase drastically when considering timescales beyond the twenty-first century. In fact, the Intergovernmental Panel on Climate Change (IPCC) recently assessed that 7–14 m of global mean sea-level contribution from Antarctica by 2300 cannot be ruled out^5,63^ especially because of the structural uncertainties posed by the MICI.

Owing to the long response times of the Antarctic Ice Sheet, some changes might be triggered in the coming decades, which then unfold over much longer timescales of centuries to millennia. The effective long-term change can be quantified as sea-level commitment^3,4,73–75^. Here we focus on these long-term consequences of global warming, assessing the equilibrium response and potential critical thresholds for different drainage basins. Theory and modelling suggest that the Antarctic Ice Sheet displays hysteresis behaviour, meaning that changes can become practically irreversible and the initial ice volume cannot be regained even if temperatures were reversed^76^. For instance, the West Antarctic Ice Sheet would probably not regrow to its modern extent unless temperatures were to be at least one degree lower than pre-industrial levels.

**Fig. 1:**
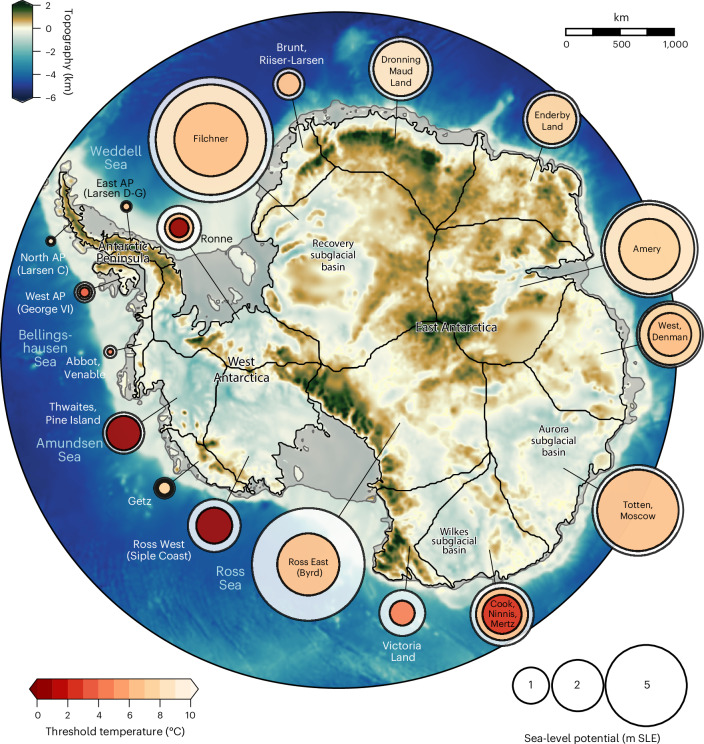
Risk map of Antarctic ice catchment basins. Map of Antarctica showing the 18 ice-sheet drainage basins as used in this analysis (thin black lines; ref. ^84^) as well as their sea-level potential (in metres sea-level equivalent, m SLE), illustrated by the size of the respective circles. Nested circles show the critical temperature levels at which the strongest ice loss occurs in the model simulations (circle colour) as well as the fraction of ice volume lost in the long term upon transgression of those thresholds with respect to the initial ice volume of the basin (circle size). Background shading shows the bedrock topography (tan–brown above sea level, white–blue below sea level); ice shelves are highlighted by grey shading. AP, Antarctic Peninsula. Observed Antarctic topography from the Bedmap2 dataset (ref. ^95^).

Owing to the heterogenous topography of Antarctica as well as the varying atmospheric and oceanic conditions, this overall hysteresis behaviour could, however, comprise or even obscure individual tipping points in different ice-sheet drainage basins. In this study, we disentangle the dynamic response for 18 individual drainage basins (Methods; Fig. 1) to quantify potential thresholds and the respective contributions to global sea-level rise and to reveal the respective key driving processes. We then assess the risk—here understood as a combination of critical threshold temperature and the corresponding impact through sea-level rise—revealing the most critical basins that warrant particular attention in monitoring and future research.

## Long-term simulations of Antarctic ice loss per basin

Here we identify the dynamic regimes and critical temperature thresholds leading to large-scale long-term ice loss in the drainage basins of Antarctica using the fully dynamic Parallel Ice Sheet Model^77,78^ (PISM). To assess the inherent (long-term) stability behaviour of the ice sheet, we apply a methodology which has been adopted previously to study the stability of some of the major climate components of Earth—among them, the Atlantic Meridional Overturning Circulation^79–81^, the Greenland Ice Sheet^82^, or the Antarctic Ice Sheet as a whole (not considering the per-basin contributions)^76^. The applied methodology consists of idealized warming experiments, in which, starting from an equilibrium reference ice-sheet state, the global mean temperature—defined here as the globally averaged surface air temperatures over land and ocean—is incrementally ramped up until complete deglaciation of all ice basins is achieved (Methods). As the applied warming rate is slower than the typical ice-sheet response times, it is ensured that the system is able to follow the change while at the same time remaining as close as possible to equilibrium. At each full degree, this quasi-static simulation is extended under fixed global mean temperature levels, until volume changes become negligible and the ice sheet reaches a steady state. While computationally more expensive than, for example, often-used step-forcing experiments (which could introduce abrupt transient effects), this approach ensures that we can identify critical thresholds or tipping points systematically, as the quasi-static state changes are an important prerequisite for a true stability assessment. This complements simulations which are based on faster transient forcing and provides more fundamental understanding to put, for instance, the response of the ice sheet to global warming overshoot scenarios into context.

**Extended Data Fig. 1:**
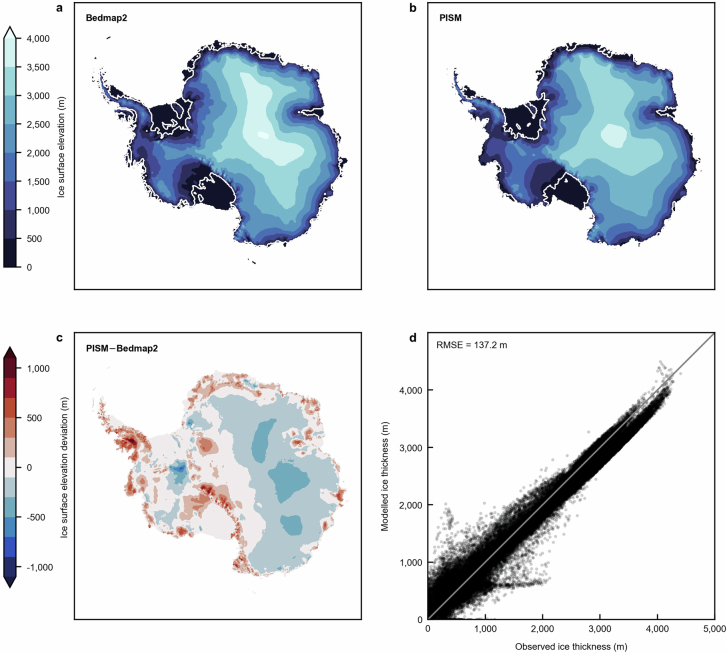
Comparison of modelled and observed ice geometry. **a**, Observed Antarctic ice surface elevation from the Bedmap2 dataset (ref. ^95^), regridded to 16 km. Grounding lines are shown in white. **b**, Modelled ice surface elevation of the reference equilibrium state serving as initial configuration for the experiments. Grounding lines are shown in white. **c**, Difference between modelled and observed ice surface elevations. **d**, Scatter plot comparing modelled and observed ice thickness for each model grid cell. The grey line illustrates where modelled ice thickness would perfectly match the observations. RMSE, root-mean-square error.

**Extended Data Fig. 2:**
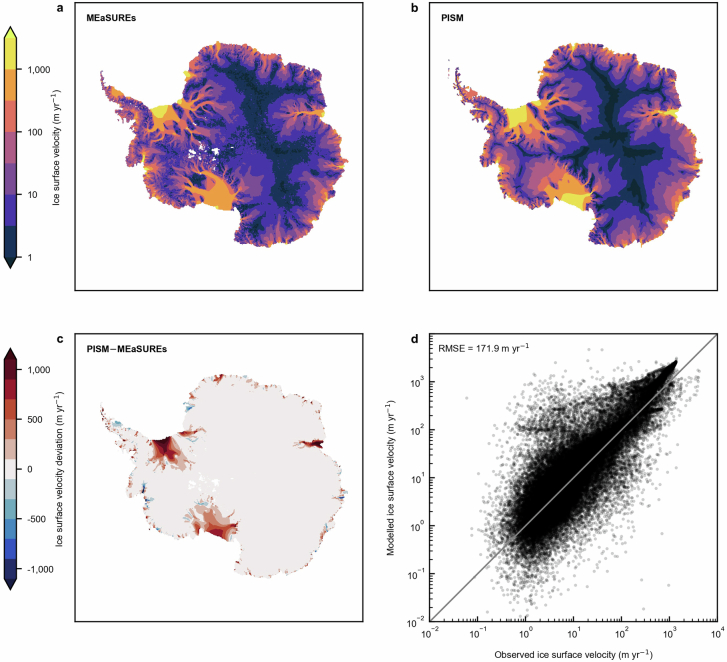
Comparison of modelled and observed ice velocities. **a**, Observed Antarctic ice surface velocities derived from multiple satellite interferometric synthetic-aperture radar systems as part of the MEaSUREs dataset (ref. ^112^), regridded to 16 km. **b**, Modelled ice surface velocities of the reference equilibrium state serving as initial configuration for the experiments. **c**, Difference between modelled and observed ice surface velocity. **d**, Scatter plot comparing modelled and observed ice surface velocities for each model grid cell. The grey line illustrates where modelled ice surface velocities would perfectly match the observations. RMSE, root-mean-square error.

We start our simulations from a reference equilibrium state closely resembling the pre-industrial Antarctic Ice Sheet configuration (Methods; Extended Data Figs. 1 and 2). Note that, while the steady state is prerequisite for our methodology, observations show that the Antarctic Ice Sheet has been subject to notable changes over the last decades as a consequence of anthropogenic climate change and isostatic rebound following the last glacial termination, suggesting that the ice sheet is not in equilibrium any longer^27,83^. However, since the observational data which are needed to form the geometric and climatic boundary conditions for the simulations are only available from the second half of the twentieth century, we interpret the pre-industrial ice-sheet configuration as the closest analogue to this equilibrium state. Any temperature anomalies are therefore taken with respect to pre-industrial levels. The ice drainage basin boundaries used in the analysis are based on the Antarctic drainage divides developed by ref. ^84^ (Methods; Extended Data Table 1).

## Gradual decline versus tipping dynamics

**Fig. 2:**
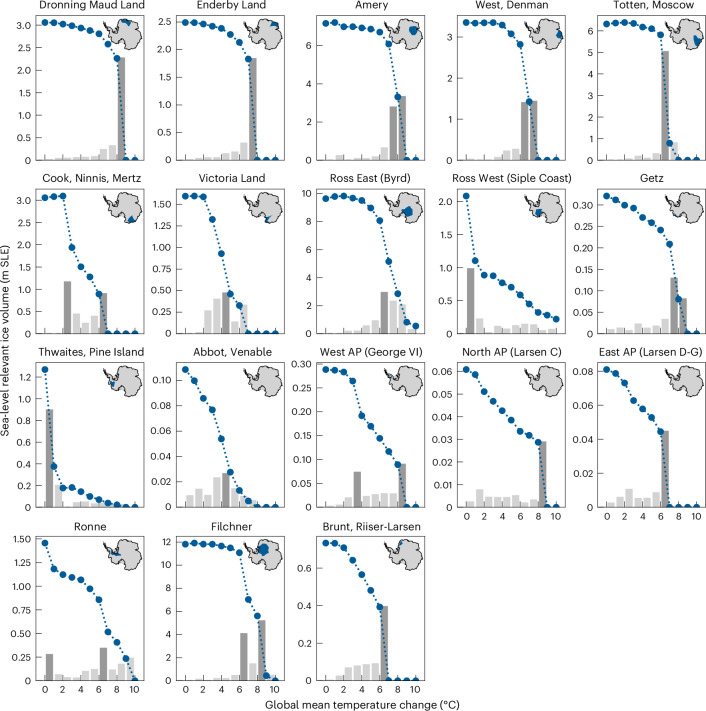
Gradual decline versus tipping dynamics of Antarctic ice basins with global warming. For each ice basin, blue dots show the initial (at pre-industrial temperature levels) sea-level relevant ice volume (Methods) and the remaining steady-state ice volume for each global warming level. Grey histograms indicate the long-term ice loss per degree of warming, with dark grey bars marking the strongest decline for the basin. Inset maps show the location of the basin within Antarctica^84^.

**Extended Data Fig. 3:**
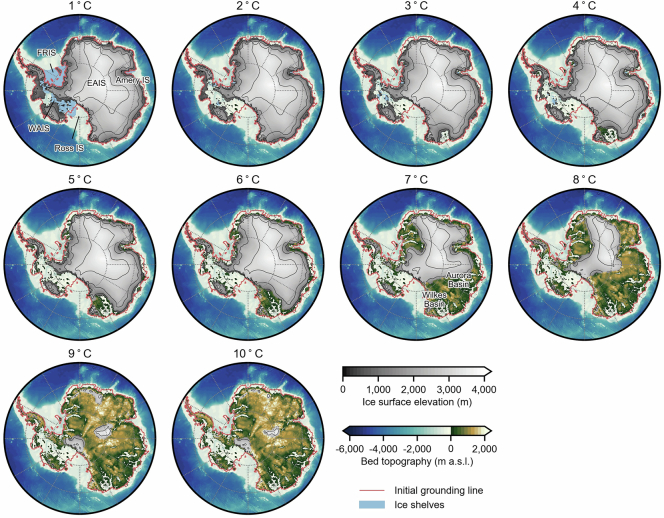
Equilibrium ice-sheet geometries under different warming levels. Equilibrium ice-sheet surface elevations (in metres) for different warming levels (global mean temperature anomaly above pre-industrial level). Ice surface-height contours are delineated at 1,000-m intervals. Grounding-line locations of the pre-industrial reference equilibrium state are shown in red; ice shelves are highlighted in light blue. Blue shadings illustrate the bedrock depth in metres below the present-day sea level; brown shadings illustrate the bedrock elevation in metres above the present-day sea level (m a.s.l.) after full glacial isostatic rebound. EAIS, East Antarctic Ice Sheet; FRIS, Filchner–Ronne Ice Shelf; IS, ice shelf; WAIS, West Antarctic Ice Sheet.

The (quasi-)equilibrium states that the Antarctic Ice Sheet approaches at increasing levels of global warming (Extended Data Fig. 3) reveal qualitatively different modes of ice-sheet response to warming for different regions of the ice sheet. To disentangle these different dynamic responses, we examine the relative ice volume loss per degree of warming for each ice-sheet drainage basin. Despite the linear increments in temperature forcing, the response in many cases is strongly nonlinear and exhibits steps and distinct peaks for all 18 drainage basins (Fig. 2, grey bars). Overall, the ice response can be broadly categorized into either a rather gradual ice volume loss with warming (here termed ‘gradual decline’) or a threshold-type response (‘tipping dynamics’). Examples for the first case are Abbot/Venable, West Antarctic Peninsula (George VI) and Ronne, which lose their ice volume almost linearly or in several increments with warming (Fig. 2 and Extended Data Table 2). Examples for drainage basins exhibiting a nonlinear tipping point behaviour are Dronning Maud Land, Enderby Land, Amery, West/Denman, Totten/Moscow and Filchner in East Antarctica, as well as the Thwaites/Pine Island basin in West Antarctica. For these drainage basins, only small changes occur throughout a relatively large warming range, followed by abrupt and large-scale ice volume decline thereafter, with near-complete volume loss at only very little additional warming. This also suggests that the ice loss in these basins is mainly driven by dynamic instability mechanisms, such as MISI or the surface melt–elevation feedback.

The remaining Antarctic drainage basins show a combination of gradual decline and tipping dynamics. For example, Ross West (Siple Coast), Cook/Ninnis/Mertz and Victoria Land show abrupt change around ~1–3 °C of global warming above pre-industrial levels and gradual decline at higher warming levels. In contrast, Brunt/Riiser-Larsen, Ross East (Byrd), Getz, North Antarctic Peninsula (Larsen C) and East Antarctic Peninsula (Larsen D–G) show a gradual decline for lower warming levels but exhibit notable threshold behaviour at warming levels exceeding 6 °C above pre-industrial levels.

**Fig. 3:**
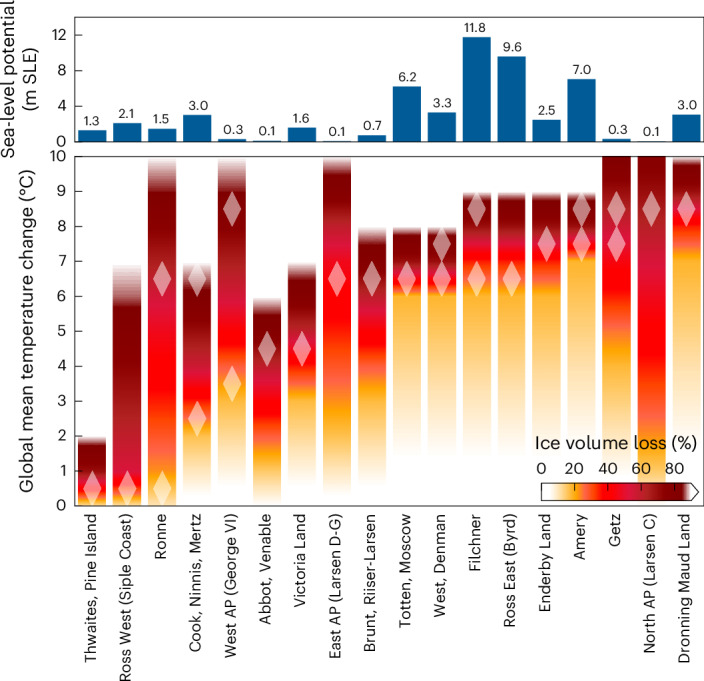
Critical temperature thresholds for Antarctic ice basins. Bottom panel: burning embers show—for each of the 18 Antarctic ice basins—the percentage of long-term (equilibrium) sea-level relevant ice volume loss compared with the respective initial ice volume, at different levels of global warming (in °C above pre-industrial temperature levels, interpolated between full degrees). White diamonds mark the one-degree temperature interval of the strongest decline (ice loss per degree of warming, see also Fig. 2). In some basins, two critical temperatures yielding peak volume loss are found—this can be interpreted as the respective basin having two tipping points. Top panel: sea-level potential for each basin, given by the initial modelled sea-level relevant ice volume in metres sea-level equivalent.

To identify in which one-degree temperature interval the highest relative ice loss for each basin occurs, we here identify critical temperature levels based on peak prominence (Fig. 2, dark grey bars). Here we only consider those peaks in ice loss where the prominence exceeds a minimum fraction of 15% of the initial ice volume of the basin. Note that these critical temperature levels can either be tipping points (their transgression causing abrupt ice loss) or simply the levels for which the highest ice loss occurs for a particular basin. In fact, we find that this 15% significance mark is transgressed in all basins at least once. In case of a double peak (for example, for Amery, West/Denman and Getz), we identify the entire two-degree temperature interval as critical. The Ronne, Filchner, Cook/Ninnis/Mertz and West Antarctic Peninsula (George VI) basins exhibit even two distinct thresholds (Figs. 1–3).

## Critical thresholds for ice basin stability

**Fig. 4:**
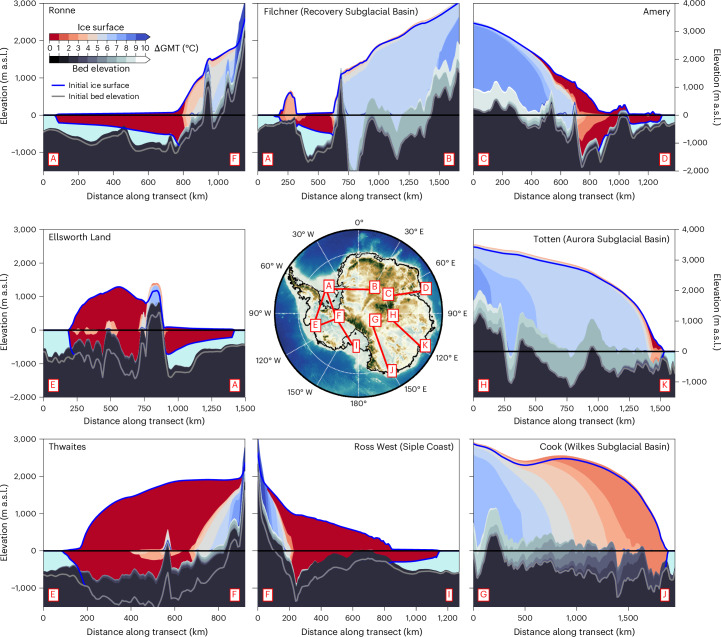
Retreat of ice fronts with increasing warming. Colour shadings (red–blue) show the modelled equilibrium ice extent (elevations in metres above sea level, m a.s.l.) for different global warming levels (ΔGMT in °C, starting from the initial, pre-industrial state, shown as blue line) along the transects indicated in the central map panel. Black–grey colour shadings indicate the respective changes in the bedrock topography as it lifts up with reducing ice load (the initial, pre-industrial bedrock topography is shown as a grey line). Observed Antarctic bed topography shown in the central map panel from the Bedmap2 dataset (ref. ^95^).

Of all Antarctic regions, the West Antarctic Thwaites/Pine Island, Ronne and Ross West (Siple Coast) basins are closest to their tipping points. With the lowest temperature threshold among all regions covered in our analysis, these basins are already at risk of substantial long-term ice loss below +1 °C of global warming above the pre-industrial reference temperature. At present, global mean warming has reached ~1.3 °C above the average temperatures of the second half of the nineteenth century. This suggests that these West Antarctic basins may already have passed a critical point at warming levels of the present, which could lead to their eventual disintegration (noting that this could take centuries to fully unfold)^60,85^. In the case of the Thwaites/Pine Island basin, this hypothesis has raised concern for several years^55–58^ and is consistent with ample observations reporting the highest ice loss from this region over the past decades^26,51,86–89^. We find that, upon transgression of its threshold, the Thwaites/Pine Island basin is committed to the loss of about 70% of its initial (that is, pre-industrial reference) sea-level relevant volume, translating into 0.9 m of long-term sea-level rise in the +1 °C equilibrium simulation. In this case, the ice loss is mainly caused by the onset of MISI^90,91^, with large-scale unstable retreat of the grounding lines occurring on the retrograde sloping bed portions, eventually slowing down on the prograde sloping bed regions (Fig. 4). The slow-down and subsequent stabilization can be supported by strong glacial isostatic rebound following the large-scale retreat^92–94^.

For global warming levels between 2–3 °C, the Cook/Ninnis/Mertz basin in East Antarctica (associated with the Wilkes Subglacial Basin) is at risk of potentially losing 40% of its initial ice volume above flotation over time, resulting in a long-term rise in global mean sea level by about 1.2 m. Putting this in relation to the future scenarios (SSPs) commonly used, for example, in the IPCC, such warming levels are reached by the end of this century in all scenarios except for the most optimistic one (SSP1-1.9, ref. ^5^; note, however, that we are here considering the equilibrium response to fixed climate conditions to assess the long-term stability of the ice basins and only refer to SSPs for context). The underlying mechanism, where a comparably small perturbation at the Cook/Ninnis/Mertz basin outlet glaciers (coastal ‘ice plug’) can trigger long-term, self-amplified retreat has been previously investigated in model simulations^61^. A second threshold between 6–7 °C in this basin may lead to further loss of about 30% of its initial sea-level relevant volume and an additional contribution to sea-level rise of approximately 0.9 m. Owing to their dynamic connection, parts of the adjacent Victoria Land basin drain into the Southern Ocean through Wilkes Subglacial Basin once global warming levels between 4–5 °C are exceeded, leading to about 0.5 m of additional sea-level rise contribution.

Also in West Antarctica (for example, Abbot/Venable) and along the West Antarctic Peninsula (George VI) we find critical global warming levels between 3–5 °C above pre-industrial levels. Note that such warming levels could be reached by the end of the twenty-first century when following the IPCC emission scenario SSP2-4.5 and all higher scenarios (very likely range, ref. ^5^).

For global warming levels beyond 6 °C above pre-industrial levels, we find threshold behaviour in almost all regions in East Antarctica (that is, Filchner, Brunt/Riiser-Larsen, Dronning Maud Land, Enderby Land, Amery, West/Denman, Totten/Moscow, Cook/Ninnis/Mertz and Ross East (Byrd)), corresponding to a combined sea-level commitment of more than 26 m. Further critical thresholds are also found for Getz basin in West Antarctica and along the Antarctic Peninsula. In addition, Ronne and Cook/Ninnis/Mertz basins display a second critical threshold between 6–7 °C and Filchner basin a second threshold between 8–9 °C. Note that, in particular, the Getz and the Antarctic Peninsula basins are very small in terms of sea-level potential compared with the other basins and further small ice caps remaining at high altitudes for even higher temperatures also result in a threshold signal, but one that is dynamically less meaningful. Above +10 °C of global warming above pre-industrial levels, we find that Antarctica becomes virtually ice free in the long-term (as already shown in ref. ^76^; these very high warming levels are here only added for completion).

## Risk assessment

Overall, the West Antarctic basins prove to be most vulnerable, large parts of which are at risk of crossing critical thresholds, potentially as low as 1–2 °C of global warming. In fact, about 40% of the present West Antarctic sea-level relevant ice volume (approximately 2.1 metres sea-level equivalent (m SLE)) is committed at this warming level. While several of the critical thresholds for East Antarctic basins are found beyond 6 °C of warming above pre-industrial levels, some significant thresholds occur between 2 °C and 5 °C (Figs. 1–3).

**Fig. 5:**
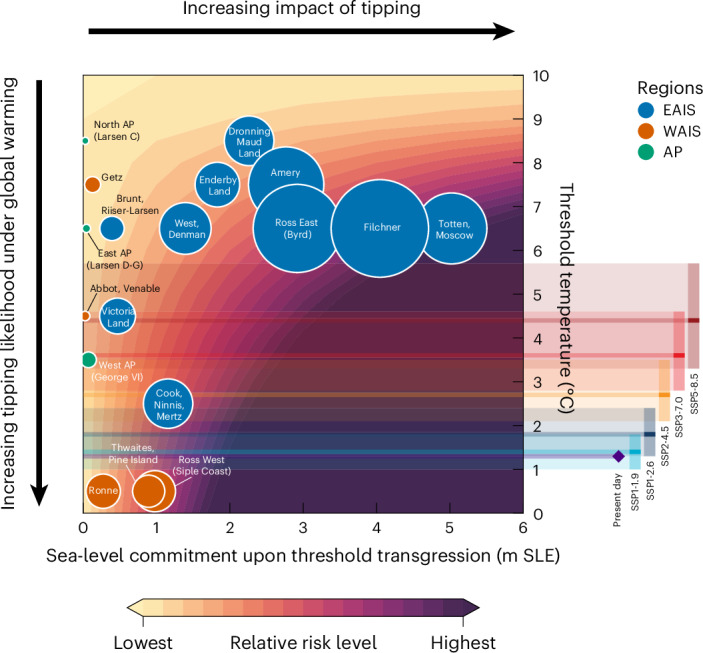
Risk assessment of critical threshold temperatures and respective sea-level commitments. Shown for each Antarctic ice catchment basin is the critical threshold temperature (in °C of global warming above pre-industrial levels), that is, the one-degree interval in which the strongest ice loss occurs, versus the sea-level commitment of the tipped basin, given by the respective equilibrium ice volume loss within the critical temperature interval (metres sea-level equivalent, m SLE). The size of the circles corresponds to the initial ice volume of the basin and the colour to the ice-sheet region. The background shading denotes the associated relative risk level, given as the product of tipping likelihood (distance from threshold temperature) and tipping impact (sea-level commitment from tipping). Coloured bars on the right show the projected global mean surface temperature warming in 2081–2100 relative to the pre-industrial period, given by the median (dark) and 5–95% confidence interval (light), respectively, for five illustrative IPCC emission scenarios^5^. The observed present-day warming is shown in purple. AP, Antarctic Peninsula; EAIS, East Antarctic Ice Sheet; WAIS, West Antarctic Ice Sheet.

In a combined risk analysis, we assess the critical warming levels together with the respective long-term ice loss caused when crossing said critical warming levels, as well as the (present-day) total sea-level potential for each basin (Fig. 5). Together, the critical warming levels and respective committed sea-level impacts give a valuable first-order indicator for the risk level of a drainage basin, also in light of adaptation planning.

Among the basins with the highest risks are Totten/Moscow, Filchner and Ross East (Byrd)—mainly because of their high sea-level commitment—and Thwaites/Pine Island, Ross West (Siple Coast) and Cook/Ninnis/Mertz (Wilkes Subglacial Basin)—mainly because of their respective low critical warming thresholds. Irrespective of this risk classification, however, it is important to keep in mind that substantial sea-level rise can occur even before reaching the critical warming levels identified here and that any sea-level commitment would have severe impacts on coastal ecosystems, infrastructures and populations^2,5^.

Importantly, while our analysis highlights the potential critical thresholds for each basin, our analysis is not to be misunderstood as sea-level projections, but rather an overarching stability analysis of the Antarctic Ice Sheet and the respective dynamic response of each basin (gradual decline versus tipping dynamics).

**Extended Data Fig. 4:**
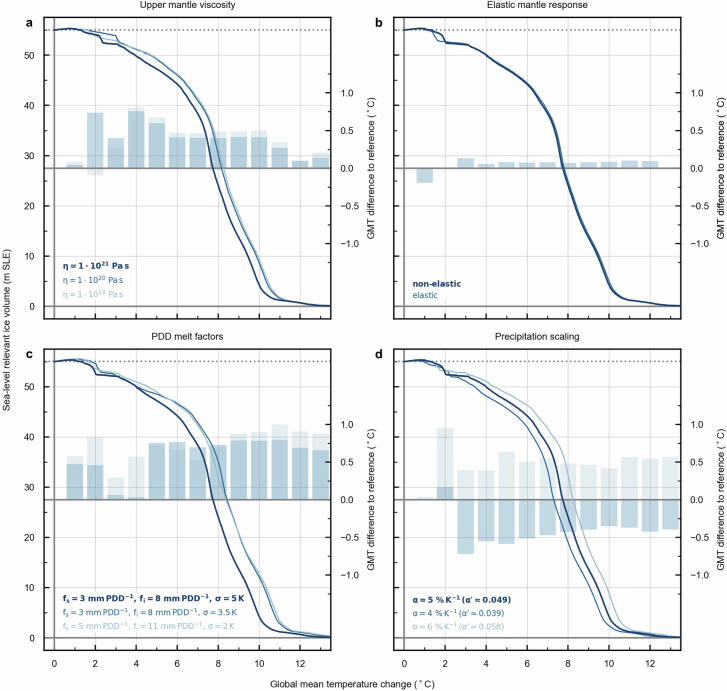
Model sensitivity to parameter variations (based on the quasi-static experiment). Sea-level relevant ice volume (in metres sea-level equivalent, m SLE) as a function of global mean temperature (GMT) change, based on the quasi-static experiment (using a warming rate of 0.0001 °C yr^−1^ above pre-industrial levels), showing the model sensitivity to various model parameters. The thick dark blue line shows the reference simulation. **a**, Sensitivity to variations in upper mantle viscosity η in the Earth-deformation model. **b**, Sensitivity to including the elastic component of the Earth-deformation model (elastic vs. non-elastic response). **c**, Sensitivity to parameters of the positive degree-day (PDD) surface melt scheme, varying the melt coefficients for snow *f*_*s*_ and ice *f*_*i*_, and standard deviation of the PDD distribution σ. **d**, Sensitivity to the precipitation scaling factor α that relates precipitation changes to atmospheric temperature and ice surface elevation changes, respectively. In all panels, the light blue bars (with respect to the right-hand axis) indicate, for each full degree of warming, the temperature difference – that is, the ‘shift’ in GMT – relative to the reference simulation yielding the same ice volume for the respective parameter variation (in °C).

**Extended Data Fig. 5:**
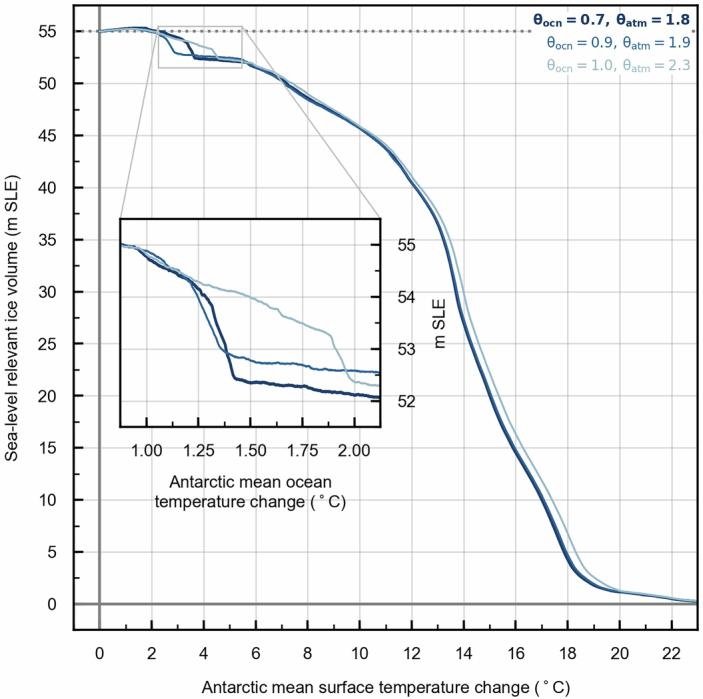
Global to regional Antarctic temperature conversion (based on the quasi-static experiment). Sea-level relevant ice volume (in metres sea-level equivalent, m SLE) as a function of Antarctic mean surface temperature change, based on the quasi-static experiment (using a warming rate of 0.0001 °C yr^−1^ above pre-industrial levels), showing the model sensitivity to variations in the scaling factors used to convert global temperature changes into Antarctic regional surface air (θ_atm_) and ocean (θ_ocn_) temperature changes, derived from long-term global climate model output (ref. ^114^). The thick dark blue line represents the reference simulation. The inset shows a zoom into the lower temperature regime, with ice volumes plotted as a function of circum-Antarctic ocean temperature change, the primary driver of ice loss at low warming levels.

**Extended Data Fig. 6:**
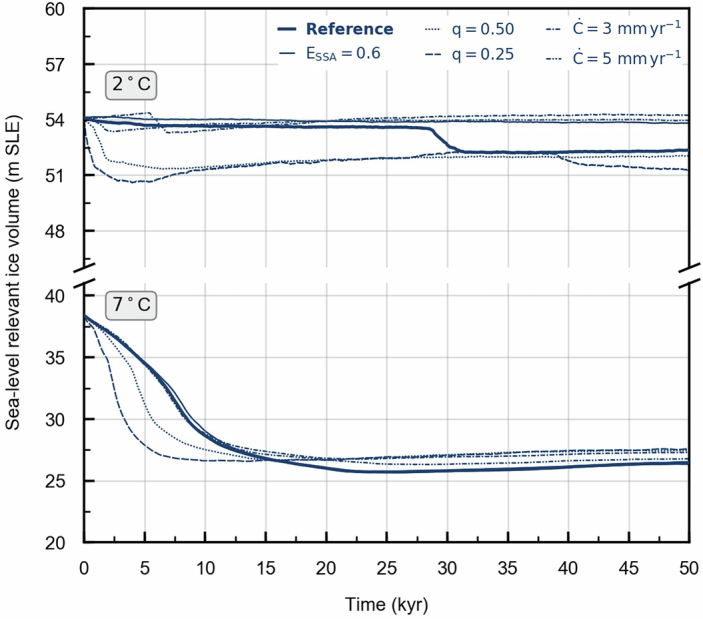
Model sensitivity to parameter variations (based on equilibrium experiments). Evolution of sea-level relevant ice volume (in metres sea-level equivalent, m SLE) in two representative sets of equilibrium simulations at 2 °C and 7 °C of global mean warming above pre-industrial levels, showing the model sensitivity to variations in critical ice-dynamical model parameters (flow enhancement factor for the SSA velocities *E*_*SSA*_; exponent in the ‘pseudo-plastic’ sliding law *q*; decay rate of the subglacial meltwater in the till layer *Ċ*) in two different representative temperature and stability regimes. The thick dark blue line shows the reference simulations.

Additional feedbacks may be missing from our analysis, including MICI which is not included in our simulations. In future research, further interactions could be considered in a fully coupled Earth system model; however, while some first simulations with Earth system models including interactive ice sheets are emerging, this is not a viable option for the type of stability analysis presented here because of the given computational constraints. For the same reason, a full quantitative uncertainty analysis covering all relevant model parameter choices across all conducted equilibrium experiments is computationally not feasible; we have, however, explored a set of parametric uncertainties (for example, regarding the global-to-regional temperature conversion, surface mass balance, glacial isostatic adjustment and crucial ice-flow processes detailed in the model description), showing that our results are qualitatively robust (Methods; Extended Data Figs. 4–6). A representative model sensitivity ensemble for 2 °C of warming shows that, while parameter variations can affect the timing of ice loss, all simulations eventually approach one of two nearby equilibrium states which emerge as a robust property. For higher warming levels, internal dynamics due to parameter choices play less of a role and ice volume converges with mere differences in the timing of ice-sheet retreat (Extended Data Fig. 6). With additional computational power in the future, the grid resolution could also be refined. However, with the primary aim of identifying critical thresholds between equilibrium states (rather than classifying the transient ice-sheet behaviour in reaching these states), the model grid resolution used here is sufficient to capture the essential feedback dynamics. So, while model grid resolution can be a decisive factor for regional grounding-line dynamics and the precise timing of marine ice loss processes in transient sea-level projections, it is only of secondary importance here.

Overall, our results indicate that the Antarctic Ice Sheet should be viewed not as one (or two) monolithic tipping element(s), but rather as a network of dynamically interacting ice basins, many—but not all—of which show the potential for nonlinear thresholds and tipping behaviour. While our study focuses on the individual drainage basins, it also shows that there needs to be a better understanding of the dynamic clusters, defined as those regions responding coherently to a given temperature. This idea can be generalized to other potential tipping elements in the Earth system, where finding the ‘right’ scale of aggregation is crucial to disentangling the dampening and reinforcing feedbacks. This is especially important in the search of potential early-warning indicators for such large-scale disruptions in the Earth system.

## Methods

### Ice-sheet model

The model simulations used for the analysis have been carried out using a modified version of the Parallel Ice Sheet Model (PISM; www.pism.io), v1.0. PISM is an open-source, high-resolution, thermomechanically coupled and polythermal ice flow model^77,78,96^ which is widely adopted in the scientific community for simulating the evolution of ice sheets and glaciers to constrain projections of future sea-level rise. The model version adopted here is the same as in ref. ^76^. In simulations over the last two glacial cycles, this model configuration has been proven capable of adequately reproducing the dynamic evolution of Antarctica across its glacial–interglacial history, resulting in simulated present-day ice-sheet configurations reasonably close to observations^97^.

PISM is a hybrid shallow ice flow model in which ice flow velocities resulting from two stress balance approximations—the shallow ice and shallow shelf approximations—are superimposed over the entire ice-sheet/ice-shelf domain^78^. This ensures smooth and consistent transitions between the different flow regimes in the interior of the ice sheet, where flow is dominated by vertical shearing, in the ice streams, which are sliding on the bed and in the floating ice shelves, where flow is characterized by fast plug flow.

The simulations are performed using a regular rectangular grid with 16-km horizontal resolution. The vertical grid spacing is quadratic, ranging from 20 m at the ice base to 100 m at the top of the computational domain. While computational feasibility of our long-term simulations puts a strong limit on model grid resolution, PISM uses a subgrid-scale linear interpolation of the basal shear stress between adjacent grounded and floating cells to ensure a free and reversible grounding-line migration, which has been shown to compare well with full-Stokes results throughout a wide range of spatial resolutions—including the resolution used here—qualitatively as well as quantitatively^98^.

The sliding of the ice sheet over the underlying bedrock is represented by a generalized pseudoplastic power law, which relates bed-parallel shear stress *τ*_b_ and basal sliding velocity *u*_b_ (ref. ^99^):

$${\tau }_{{\rm{b}}=-{\tau }_{{\rm{c}}}}\frac{{u}_{{\rm{b}}}}{{u}_{0}^{q}\,{|{u}_{{\rm{b}}}|}^{1-q}},$$

where *q* = 0.75 is the pseudoplastic sliding exponent and *u*_0_ = 100 m yr^−1^ is a reference velocity. The yield stress *τ*_c_ is determined according to the Mohr–Coulomb criterion based on the till friction angle (an heuristic shear strength parameter for the till material property) and the effective till pressure^100^. The former is iteratively optimized in the grounded-ice region to minimize the mismatch of modelled and observed ice surface elevations^97^.

Iceberg calving at the margins of the ice shelves is calculated on the basis of spreading rates (‘eigencalving’, ref. ^101^), using a proportionality factor of 10^17 ^m s. To ensure numerical stability, we further apply a minimum thickness criterion of 50 m at the calving front^102^ and remove floating ice in cells which were marked as open ocean during the initial timestep.

The glacial isostatic adjustment of the bedrock and seafloor in response to changing ice loads are modelled using a viscoelastic Earth-deformation model^103,104^, assuming a spatially uniform upper mantle viscosity of 10^21^ Pa s (ref. ^68^) and a standard density of 3,300 kg m^−3^. As we here only consider equilibrium ice-sheet states, we find only little sensitivity of our results to variations in Earth model parameters. Lower mantle viscosities and lower flexural rigidity (thinner elastic lithosphere), however, can slow transient ice-sheet retreat and shift thresholds to slightly higher (<1 °C) temperatures (Extended Data Fig. 4a,b).

### Climatic boundary conditions

At the ice–atmosphere boundary, we compute melt and runoff using a positive degree-day (PDD) scheme with melt coefficients of 3 mm PDD^−1^ and 8 mm PDD^−1^ for snow and ice, respectively, and a 5 °C standard deviation to account for diurnal cycles and synoptic variability, applying a sinusoidal yearly temperature cycle^105,106^. Higher melt factors or lower standard deviation lead to more ice loss for the same temperature forcing or to the same ice loss at lower temperatures. However, for a plausible range of uncertain PDD parameters, the corresponding shift on the temperature scale remains likely within the one-degree resolution of our analysis (Extended Data Fig. 4c).

Annual and summer mean surface air temperatures are thereby parameterized as a function of latitude and surface elevation^97^, based on multiple regression analysis of ERA-Interim data^107^. This allows the temperature field to adjust to a changing geometry by deploying a prescribed atmospheric temperature lapse rate *Γ* of −8.2 K km^−1^.

Surface accumulation is derived from the Regional Atmospheric Climate Model (RACMO v2.3p2; ref. ^108^) precipitation output, averaged over the period 1986–2005. Similar to the temperature parameterization, we introduce a climatic correction for precipitation as a modification from PISM v1.0. By scaling the reference precipitation pattern *P*_ref_ with both the applied surface temperature anomaly and the model ice surface elevation change, this correction ensures that accumulation rates increase under warmer atmospheric conditions as expected from the Clausius–Clapeyron relationship and that geometrical changes have an influence on local precipitation through their effect on local surface temperatures^97^. The scaling of precipitation with respect to surface temperature change Δ*T* is exponential^34^:

$$P(\Delta T)={P}_{{\rm{ref}}}\times \exp ({\alpha }^{{\prime} }\times \Delta T),$$

with the exponential factor *α*′ = ln(1.05) ≈ 0.049 K^−1^ corresponding to a sensitivity *α* of 5% K^−1^ precipitation increase per degree of atmospheric warming under the assumption of a linear relationship for lower warming regimes, consistent with ref. ^32^. The scaling of precipitation with respect to surface elevation change Δ*h* is also exponential^97^, where the exponential factor *α* × *Γ* corresponds to about 51% increase in precipitation per kilometre of elevation lowering and about 34% decrease in precipitation per kilometre of elevation gain:

$$P(\Delta h)={P}_{{\rm{ref}}}\times \exp (\alpha \times \Gamma \times \Delta h).$$

The range of the sensitivity factor $\alpha$ found among Coupled Model Intercomparison Project Phase 6 (CMIP6) models is 5.46 ± 0.87% K^−1^ (Table A1 in ref. ^34^). Assuming an uncertainty of ±1% K^−1^ around our value of 5% K^−1^, we find slight shifts in the transient (quasi-static) response for a given temperature forcing (about ±0.5 °C), with thresholds still to be found in the same one-degree temperature intervals (Extended Data Fig. 4d).

Subshelf melting at the ice–ocean boundary underneath the ice shelves is computed using the Potsdam Ice-shelf Cavity Model (PICO; ref. ^109^). We drive PICO with observed ocean temperature and salinity data from ref. ^110^, averaged over the period 1975–2012. For the overturning strength coefficient and turbulent heat exchange velocity across the ice–ocean boundary we adopt parameter values of 0.5 Sv (kg m^−3^)^−1^ and 10^−5^ m s^−1^, respectively. These values are the same as in refs. ^76,111^ and slightly lower than the values used in refs. ^60,109^, resulting in a slightly conservative estimate of subshelf melt rates.

### Reference ice-sheet state

We start the model simulations from a reference equilibrium ice-sheet state that resembles the pre-industrial Antarctic Ice Sheet geometry as closely as possible. This reference equilibrium state is based on an equilibrium state generated as part of the initial state model intercomparison activity focusing on Antarctica (initMIP-Antarctica; ref. ^111^) within the framework of the Ice Sheet Model Intercomparison Project for CMIP6 (ISMIP6), the primary CMIP6 activity focusing on the Greenland and Antarctic ice sheets. It was initialized from Bedmap2 geometry^95^, with surface accumulation from RACMO v2.3p2 (ref. ^108^), observed ocean temperature and salinity data from ref. ^110^ to drive PICO and run over 100 kyr (for more details, see Appendix B12 of ref. ^111^). Using our modified PISM version^76^, we extended this initMIP equilibrium simulation for another 150 kyr under the same climatic boundary conditions. In comparison to the model configuration used in ref. ^111^, in addition to some minor model updates and fixes, the modified model version accounts for glacial isostatic adjustment of the bedrock and adopts parameterizations of surface air temperature and precipitation that dynamically account for changes in ice-sheet geometry, with surface melt rates being now computed by a PDD scheme (see above and ref. ^76^ for details). A comparison of the model reference equilibrium state at the end of the 250-kyr spin-up with observational data of ice geometry^95^ and velocities^112^ is shown in Extended Data Figs. 1 and 2, respectively.

In light of recent observations, which show that the Antarctic Ice Sheet has undergone notable changes in recent decades due to anthropogenic climate forcing, we note that the ice sheet is probably no longer in equilibrium^27,83^. However, as observational data—which constrain the geometric and climatic boundary conditions in our model simulations—are only available from the second half of the twentieth century, we interpret the pre-industrial ice-sheet configuration as the closest analogue to this reference equilibrium state and therefore take temperature anomalies in all experiments presented here with respect to pre-industrial (~1850–1900) levels.

### Warming scenarios

Starting from the reference equilibrium ice-sheet state, we deploy generic warming scenarios to assess the long-term stability behaviour and critical thresholds of the ice sheet in response to changing global temperatures. In these scenarios, a spatially uniform global mean temperature anomaly that is gradually increasing over time is applied to the boundary climate in the model until near-complete deglaciation of the entire ice sheet is achieved. The rate of change of the temperature increase of 0.0001 °C yr^−1^ is thereby slower than the typical response timescale of the ice sheet to ensure that the system can follow the change while remaining close to equilibrium at all times (‘quasi-static’ change). These simulations are then extended at each full degree under fixed global mean temperature levels until a real steady state is reached, that is, volume changes in response to the forcing have become negligible. Equilibrium simulations are run for at least 20 kyr and in higher warming regimes (above 6 °C of warming) for 50 kyr to account for the higher sensitivities, that is, the amount of ice loss per degree of warming.

Global mean temperature anomalies are translated into Antarctic regional (south of 66° S) atmospheric and intermediate-depth (500–2,500 m) oceanic temperature changes using constant scaling factors of 1.8 and 0.7, respectively, which are uniformly applied across the entire model domain and are derived from long-term, near-equilibrium simulations with the coupled climate model ECHAM5/MPIOM following an abrupt fourfold increase in CO_2_ (ref. ^113^). Despite different equilibrium climate sensitivities, we estimate similar scaling factors from similar long-term experiments with other climate models (MPIESM1.1, CESM1.0.4 and GISS-E2-R) participating in LongRunMIP^114^, with scaling ratios with respect to global mean temperature ranging between 1.8 to 2.3 and 0.7 to 1.0 for Antarctic regional atmospheric and oceanic temperature changes, respectively. Importantly, the ratio of Antarctic regional oceanic and atmospheric temperatures remains consistently between 0.4 and 0.5 across all analysed models. Sensitivity simulations based on the (transient) quasi-static experiment show that our results remain overall robust with respect to changes in the scaling factors within these ranges (Extended Data Fig. 5). Note that using regional scaling factors could also affect the timing of crossing respective thresholds, but the critical warming levels relevant for the equilibrium response are likely to be consistent with the ones derived here using the uniform scaling factors.

### Basin analysis

The catchment basin boundaries used in the analysis are derived from the Making Earth System Data Records for Use in Research Environments (MEaSUREs) Antarctic Boundaries for IPY 2007–2009 from Satellite Radar (v2) dataset^84^ (Extended Data Table 1), using the most recent refinements developed for the latest Ice Sheet Mass Balance Intercomparison Exercise (IMBIE-3). The basin boundaries are defined on the basis of historical nomenclature plus modern digital elevation model^95^ and ice velocity data^112^ and adjusted to match the drainage boundaries of the major ice shelves.

### Sea-level relevant ice volume

In our analysis we derive volume changes of the Antarctic Ice Sheet from changes in the ice thickness and bed elevation and convert these changes into units of metres sea-level equivalent (m SLE). This conversion assumes that only the grounded ice above flotation contributes to sea-level changes. For better comparison with previous studies (for example, refs. ^38,115^), we here follow the definition of ‘volume above flotation’ (*V*_af_), applied to the projected domain of the individual Antarctic ice basins. Our calculation is in line with the definition of *V*_af_ given in ref. ^116^ (corrected equation 1, using an ocean area of 3.61 × 10^14^ m^2^). However, we have not used the additional suggested corrections by refs. ^116,117^, that intend to account for the changing density of the melted ice in the ocean, or for the effect of glacial isostatic adjustment on the sea-level contribution (often associated with the ‘water expulsion effect’). While the implied corrections can in places be substantial, the way of converting ice volume changes does not change the qualitative behaviour and threshold temperatures in our analysis.

## Online content

Any methods, additional references, Nature Portfolio reporting summaries, source data, extended data, supplementary information, acknowledgements, peer review information; details of author contributions and competing interests; and statements of data and code availability are available at 10.1038/s41558-025-02554-0.

## Data Availability

All data used for this assessment are publicly available. Antarctic surface mass balance data from RACMO2.3p2 can be downloaded from https://www.projects.science.uu.nl/iceclimate/publications/data/2018/index.php#vwessem2018_tc. Antarctic bedrock topography and ice thickness data are from the Bedmap2 compilation, available at https://secure.antarctica.ac.uk/data/bedmap2/. MEaSUREs Antarctic ice surface velocities are available from the National Snow and Ice Data Center at https://nsidc.org/data/nsidc-0484/. The MEaSUREs Antarctic Boundaries dataset (IMBIE basins) is available from the National Snow and Ice Data Center at https://nsidc.org/data/nsidc-0709/. The ocean temperature and salinity dataset can be retrieved at https://www.geomar.de/en/staff/fb1/po/sschmidtko/southern-ocean/. The PISM model output data generated and analysed in this study are available via Zenodo at 10.5281/zenodo.17466786 (ref. ^118^).

## Extended data

**Extended Data Table 1:** Antarctic ice drainage basins

Antarctic ice drainage basin	No. (IMBIE)	Subregion (IMBIE)	Region (IMBIE)
Dronning Maud Land	2	A-A’	East Antarctica
Enderby Land	3	A’-B	
Amery	4	B-C	
West, Denman	5	C-C’	
Totten, Moscow	6	C’-D	
Cook, Ninnis, Mertz	7	D-D’	
Victoria Land	8	D’-E	
Ross East (Byrd)	9	E-E’	
Ross West (Siple Coast)	10	E’-F	West Antarctica
Getz	11	F-G	
Thwaites, Pine Island	12	G-H	
Abbot, Venable	13	H-H’	
West AP (George VI)	14	H’-I	Antarctic Peninsula
North AP (Larsen C)	15	I-I’’	
East AP (Larsen D–G)	16	I’’-J	
Ronne	17	J-J’’	West Antarctica
Filchner	18	J’’-K	East Antarctica
Brunt, Riiser-Larsen	19	K-A	

The 18 Antarctic ice drainage basins used in the analysis as well as their corresponding subregion number, subregion, and associated Antarctic regions, as defined by ref. ^84^ for the Ice Sheet Mass Balance Inter-comparison Exercise (IMBIE). AP, Antarctic Peninsula.

**Extended Data Table 2:** Critical temperature thresholds for Antarctic ice basins

Antarctic ice basin	10 %	20 %	25 %	50 %	75 %	90 %
Dronning Maud Land	6	8	8	9	9	10
Enderby Land	6	7	7	8	8	9
Amery	7	8	8	8	8	9
West, Denman	6	7	7	7	8	8
Totten, Moscow	7	7	7	7	7	8
Cook, Ninnis, Mertz	3	3	3	4	4	7
Victoria Land	3	4	4	5	6	7
Ross East (Byrd)	6	7	7	8	9	9
Ross West (Siple Coast)	1	1	1	1	5	9
Getz	3	5	6	8	8	–
Thwaites, Pine Island	1	1	1	1	2	2
Abbot, Venable	1	2	2	4	5	6
West AP (George VI)	3	4	4	6	9	10
North AP (Larsen C)	1	2	3	7	–	–
East AP (Larsen D–G)	2	3	3	8	9	10
Ronne	1	1	1	6	8	10
Filchner	7	7	7	8	9	9
Brunt, Riiser-Larsen	3	4	4	6	7	8

For each of the 18 Antarctic ice basins, the global warming level (in °C above pre-industrial) is given at which a certain percentage of the initial sea-level relevant ice volume of the basin is lost in the long term (equilibrium). AP, Antarctic Peninsula.

## Code availability

PISM is freely available as open-source code under the GPL license from www.pism.io. The code version used in this study is available via Zenodo at 10.5281/zenodo.3956431 (ref. ^119^). PISM input data are preprocessed using https://github.com/pism/pism-ais with original data citations. The Python scripts used to analyse the data and create the figures are available from the corresponding author upon reasonable request.
